# Ethnicity/race, parent educational attainment, and obesity associated with prediabetes in children

**DOI:** 10.1038/s41387-023-00244-4

**Published:** 2023-09-09

**Authors:** Reem Ghaddar, Erin A. Hudson, Matthew R. Jeans, Sarvenaz Vandyousefi, Matthew J. Landry, Jaimie N. Davis

**Affiliations:** 1 Department of Nutritional Sciences, College of Natural Sciences, University of Texas at Austin, Austin, TX USA; 2 New York University, Grossman School of Medicine, Department of Medicine, New York, USA; 3 Stanford Prevention Research Center, School of Medicine, Stanford University, Palo Alto, USA

**Keywords:** Pre-diabetes, Risk factors

## Abstract

**Background/objectives:**

Obesity and other predictors of type 2 diabetes disproportionally affect Hispanic and Black children in the US compared to non-Hispanic White (NHW) children. Yet, the prevalence of prediabetes in children remains unestablished, and guidelines for screening young children are lacking. This study examined the relationships between demographic factors and prediabetes in vulnerable youth in central Texas.

**Subjects/methods:**

This is a cross-sectional analysis of baseline data from 976 3rd–5th graders (7–12 years) who participated in TX Sprouts, a school-based gardening, nutrition, and cooking trial in 16 elementary schools serving mainly children from minority backgrounds and lower-income households. Measures collected included age, sex, ethnicity, free/reduced-priced school lunch (FRL) status, parent educational attainment (questionnaires), BMI from height (stadiometer) and weight (TANITA scale), and prediabetes status from fasting plasma glucose (FPG) and HbA1c. Regressions examined cross-sectional associations between demographics and FPG, HbA1c, and prediabetes.

**Results:**

Children were 47% male, 67% Hispanic, and 10% Black, with a mean age of 9.3 years; 71% received FRL, 50% had overweight/obesity, and 26% had prediabetes. Prediabetes rates were 2.8 and 4.8 times higher in Hispanic and Black children compared to NHW children, respectively (*p* ≤ 0.001), and 1.5 times higher in children with obesity versus normal BMI (*p* = 0.02). Children of parents with only an 8th-grade education, some high school education, or a high school degree had 3.1, 2.7, and 2.2 times higher odds of having prediabetes compared to children of college graduates, respectively (*p* ≤ 0.004). Analyses with FPG and HbA1c yielded similar results.

**Conclusion:**

These findings suggest a potential need for earlier screening, more comprehensive testing guidelines, and prevention programs tailored toward minority children, children with obesity, and children of parents with low educational attainment. Future research should explore this finding in a larger, nationally representative sample.

## Introduction

In 2014, 34% of children (6–11 years) in the US were overweight or obese. This rate was higher in Hispanic and Black children compared to non-Hispanic White (NHW) children (46% and 38% versus 29%, respectively) [1]. Pediatric overweight and obesity are serious public health concerns because children with obesity are at increased risk of having obesity in adulthood [2], which increases their risk of developing type 2 diabetes (T2D) [3].

Hispanic and Black populations in the US tend to be impacted by socioeconomic and environmental factors that can increase their risk of developing obesity and T2D, such as higher poverty rates and lower educational attainment compared to NHW populations [4–6]. In the US, Hispanic and Black individuals may be more likely to have lower educational attainment than NHW [7], and incremental increases in educational attainment are associated with decreases in poverty rates [4]. The causes of these racial and ethnic disparities are outside the scope of this paper but are an active area of research. [8, 9].

These factors have contributed to a rise in youth-onset T2D and its precursor, prediabetes [10]. A recent study found that 18% of adolescents (12–19 years) in the US had prediabetes, with higher rates in Hispanic (23%) and Black (21%) adolescents compared to NHW adolescents (15%) [11]. The rates of prediabetes in youth may be driven, in part, by puberty, which increases insulin resistance [12]. Some studies have found that puberty-driven insulin resistance subsides in most adolescents following puberty [10]. However, Hispanic and non-Hispanic Black youth have higher rates of progression from prediabetes to T2D during or following puberty, i.e., they are less likely to revert to normal glucose levels after puberty [13]. In addition, this post-puberty reversion to normal glucose tolerance is less prevalent among people with higher HbA1c in childhood [14]. There also appears to be a linear relationship between increasing BMI during puberty and progression to T2D [10].

The current American Diabetes Association (ADA) prediabetes testing guidelines for children are limited, recommending testing after age 10 years or onset of puberty (whichever occurs earlier) if the child is both overweight/obese and at least one other risk factor for diabetes, including being of Hispanic or Black ethnicity/race [15]. To date, prediabetes prevalence in the US in children under the age of 12 years has not been measured. Thus, the aim of this study was to assess prediabetes prevalence rates in children (7–12 years) from predominately minority backgrounds and low-income households in school settings and examine the relationships between potential risk factors (socioeconomic status, parent educational attainment, race/ethnicity, and BMI) and prediabetes markers. This study hypothesized that prediabetes rates would be higher in Hispanic and Black children compared to NHW children, higher in children with low versus high socioeconomic status, higher in children of parents with low compared to high educational attainment, and higher in children with overweight or obesity versus underweight or normal weight.

## Material/subjects and methods

This study used baseline data from TX Sprouts, a single-school year, cluster-randomized controlled gardening, nutrition, and cooking trial (2016–2019). Full methods and main outcomes of the TX Sprouts intervention are described elsewhere [16, 17]. TX Sprouts targeted 3rd–5th grade students from 16 elementary schools in the Austin, Texas area. To be eligible for participation, schools had to: (1) be within 60 miles of the UT at Austin campus; (2) have ≥50% Hispanic student body; (3) have ≥50% of students eligible for free or reduced-price lunch (FRL); and (4) have no existing garden or gardening program. The first 16 schools to respond were randomized into intervention (*n* = 8 schools) or delayed intervention (control; *n* = 8 schools). This trial was registered at ClinicalTrials.gov (NCT02668744). All study procedures were approved by the Institutional Review Board at UT-Austin. Informed consent was obtained from all participating parents and assent was obtained from participating students.

### Measurements

Demographics: Demographic data were collected through child and parent questionnaires. Parent questionnaires were self-administered and included questions about parent educational attainment, child race/ethnicity, and socioeconomic status (i.e., if their child received FRL at school).

BMI parameters: Study staff measured height with a free-standing stadiometer to the nearest 0.1 cm (Seca, Birmingham, UK) and weight using the Tanita Body Fat Analyzer (Tanita Corporation of America Inc, IL, USA, model TBF 300). Height and weight were used to determine BMI categories based on Centers for Disease Control and Prevention age- and sex-specific values [18].

Blood collection: Blood draws were optional and were conducted over a 1-week period at each school and took place before the start of the school day (on weekdays) and/or on Saturday mornings. Children were asked three times if they were fasting before the blood draw—twice during the check-in process and once by the phlebotomist conducting the draw. Children who were not fasting were asked to come back on another morning that week. Blood samples were placed on ice immediately after being drawn. Children were given a snack and their $20 cash incentive after their blood draw.

FPG was measured using the HemoCue Glucose 201 System (HemoCue America, Brea, CA) (waves 1–3), and HbA1c was measured using the DCA Vantage Analyzer (Tosoh Bioscience, Inc. San Francisco, CA) (waves 2 and 3). Prediabetes was defined using ADA diagnostic cutoffs (FPG value of 100–125 mg/dL and/or HbA1c value of 5.7–6.4%) [19]. The remaining blood was centrifuged, aliquoted, and frozen for future analyses.

Parents received their child’s FPG and HbA1c values within two weeks through a sealed envelope addressed to the parents and sent home from school with their child. It also included a letter stating that their child may have prediabetes/diabetes, that failure to fast could have elevated the results, and that follow-up with a physician is recommended. A list of local low-cost clinics was included for those who wanted to follow up. Parents of children with FPG and/or HbA1c values indicating diabetes were called by the study physician.

Data was managed using REDCap, normality was assessed for all continuous variables, and no transformations were necessary. After confirming all relevant assumptions, linear regressions were run to assess the relationships between all variables of interest (sex, age, race/ethnicity, FRL status, parent educational attainment, BMI category) and FPG and HbA1c levels, and binary logistic regressions were run to examine the relationship between the same variables and prediabetes status.

Based on prior research showing a significant interaction between race/ethnicity and socioeconomic status [20, 21], we included an interaction term in the linear regressions to model possible variation in the effect of race/ethnicity on FPG and HbA1c based on FRL status (as a proxy for socioeconomic status). Participants identifying “other” in race/ethnicity were excluded from this analysis due to the small sample size. Based on the interaction between race/ethnicity and FRL status in one of the linear regression models, the sample was stratified by race for a secondary binary logistic regression to explore variables of interest by each race/ethnicity. Analyses were performed using Statistical Package for Social Sciences, version 26 (SPSS Inc, Armonk, NY), R (version 4.2.0), and R Studio (version 2021.09.0+351) software, with 0.05 alpha level denoting statistical significance.

## Results

A total of 1111 children (35.4% of total sample) successfully completed the optional blood draw. Children with type 1 or type 2 diabetes (*n* = 7), hypoglycemic FPG (*n* = 1) [17], or missing demographic data (*n* = 127) were excluded. The final analytic sample included 976 children between seven and 12 years.

Table 1 presents demographic characteristics. Children were 47% male, 67% Hispanic, 17% NHW, and 10% Black (Hispanic or non-Hispanic unspecified), with a mean age of 9.3 years. Over half (57%) of parents had no college education, and 71% of children received FRL. Approximately 19% of children were overweight and 31% had obesity. Approximately 26% had prediabetes based on ADA diagnostic criteria [19].

**Table 1:** Sample characteristics of TX Sprouts participants.

	Total sample
	*n* = 976
Male	463 (47.4%)
Age (years)	9.30 ± 0.09
7 to 8	206 (21.1%)
9	350 (35.9%)
10	349 (35.8%)
11 to 12	71 (7.3%)
Race/ethnicity	
Non-Hispanic White	167 (17.1%)
Hispanic	658 (67.4%)
Black	98 (10.0%)
Other	53 (5.4%)
Free/reduced-price lunch recipient	694 (71.1%)
Parent education	
Less than 8th grade	146 (15.0%)
Finished 8th grade	106 (10.9%)
Some high school	99 (10.1%)
High school graduate/GED	201 (20.6%)
Some college/vocational school	223 (22.8%)
College graduate	201 (20.6%)
BMI category	
Underweight/normal^a^	489 (50.1%)
Overweight	187 (19.2%)
Obesity	300 (30.7%)
Prediabetes^b^	256 (26.2%)
Blood values	
Fasting plasma glucose (mg/dL)^c^	92.51 ± 9.60
HbA1c (% (mmol/mol))^d^	5.23 ± 0.26 (34 ± 2.9)

Data are *n* (%) or Mean ± SD.

^a^16 children were underweight.

^b^Based on ADA diagnostic criteria (FPG 100–125 or HbA1c 5.7–6.4%).

^c^*n* = 973 because three children did not have FPG values.

^d^*n* = 610 because year 1 did not include HbA1c testing.

Table 2 reports the relationships between the variables of interest and FPG and HbA1c found in Model 1, which did not include an interaction term. FPG levels were higher for both Hispanic (β = 2.44, 95%CI[0.66, 4.23], *p* = 0.007) and Black (β = 5.51, 95%CI[3.11, 7.90], *p* < 0.001) children compared to their NHW peers. FPG levels were higher in children whose parents did not have a college education versus those whose parents had a college degree (β = 2.75–5.64, *p* ≤ 0.006). HbA1c levels were lower in males than females (β = −0.05, 95%CI[−0.09, −0.01], *p* = 0.017). Hispanic and Black children had higher HbA1c levels than NHW children (β = 0.09, 95%CI[0.03, 0.15], *p* = 0.002, and β = 0.18, 95%CI[0.10, 0.26], *p* < 0.001, respectively). Children of parents with a partial high school education had higher HbA1c levels than children of college graduates (β = 0.12, 95%CI[0.03, 0.20], *p* = 0.007). Independent of demographics, obesity was associated with higher HbA1c levels (β = 0.09, 95%CI[0.05, 0.14], *p* < 0.001).

**Table 2:** Linear regression for the relationships between demographic variables and FPG and HbA1c for TX Sprouts participants.

	Fasting plasma glucose (mg/dL)			Fasting plasma glucose (mg/dL)			HbA1c (%)			HbA1c (%)		
	Model 1 (*n* = 973)			Model 2 (*n* = 920)^a^			Model 1 (*n* = 610)			Model 2 (*n* = 571)^a^		
	Unstd β	95% CI	*p*-value	Unstd β	95% CI	*p*-value	Unstd β	95% CI	*p*-value	Unstd β	95% CI	*p*-value
Sex												
Female	REF		–	REF	–	–	REF	–	–	REF	–	–
Male	0.50	(−0.68, 1.69)	0.404	0.24	(−0.99, 1.46)	0.705	−0.05	(−0.09, 0.01)	0.017	−0.05	(−0.09, −0.00)	0.03
Age (years)												
7 to 8	REF	–	–	REF	–	–	REF	–	–	REF	–	–
9	−0.4	(−2.01, 1.21)	0.625	−0.26	(−1.92, 1.40)	0.759	0.03	(−0.03, 0.09)	0.3	0.03	(−0.03, 0.09)	0.349
10	−0.29	(−1.90, 1.32)	0.725	−0.49	(−2.15, 1.17)	0.564	0.02	(−0.04, 0.08)	0.531	0.014	(−0.0, 0.07)	0.64
11 to 12	2.14	(−0.40, 4.69)	0.099	2.09	(−0.59, 4.78)	0.126	0.08	(−0.01, 0.17)	0.071	0.04	(−0.05, 0.13)	0.393
Race/ethnicity												
Non-Hispanic White	REF	–	–	REF	–	–	REF	–	–	REF	–	–
Hispanic	2.44	(0.66, 4.23)	0.007	4.57	(2.04, 7.10)	<0.001	0.09	(0.03, 0.15)	0.002	0.08	(−0.00, 0.16)	0.052
Black	5.51	(3.11, 7.90)	<0.001	3.046	(−1.18, 7.27)	0.158	0.18	(0.10, 0.26)	<0.001	0.13	(−0.00, 0.26)	0.054
Other	1.58	(−1.34, 4.51)	0.288	–	–	–	0.06	(−0.03, 0.15)	0.203	–	–	–
Free/reduced-price lunch (FRL)												
No	REF	–	–	REF	–	–	REF	–	–	REF	–	–
Yes	0.37	(−1.08, 1.81)	0.619	2.89	(−0.12, 5.89)	0.06	0.01	(−0.04, 0.06)	0.604	−0.04	(−0.13, 0.06)	0.446
Race/ethnicity*FRL												
Non-Hispanic White; No FRL				REF	–	–				REF	–	–
Hispanic; Yes FRL				−3.78	(−7.21, −0.35)	0.031				0.04	(−0.07, 0.15)	0.451
Black; Yes FRL				2.09	(−3.15, 7.34)	0.434				0.1	(−0.07, 0.27)	0.249
Parent education												
College graduate	REF	–	–	REF	–	–	REF	–	–	REF	–	–
Some college/vocational school	1.57	(−0.28, 3.42)	0.095	1.36	(−0.58, 3.30)	0.168	−0.02	(−0.08, 0.04)	0.43	−0.03	(−0.09, 0.03)	0.363
High school grad/GED	2.75	(0.79, 4.72)	0.006	2.09	(0.015, 4.17)	0.048	0.001	(−0.06, 0.07)	0.967	−0.01	(−0.07, 0.06)	0.865
Some high school	4.48	(2.08, 6.89)	<0.001	4.00	(1.50, 6.51)	0.002	0.12	(0.03, 0.20)	0.007	0.11	(0.02, 0.19)	0.017
Finished 8th grade	5.64	(3.25, 8.03)	<0.001	5.42	(2.95, 7.88)	<0.001	0.03	(−0.07, 0.14)	0.509	0.04	(−0.07, 0.14)	0.498
Less than 8th grade	4.46	(2.22, 6.71)	<0.001	4.29	(1.97, 6.62)	<0.001	0.04	(−0.04, 0.11)	0.345	0.04	(−0.04, 0.12)	0.276
BMI category												
Underweight/normal	REF	–	–	REF	–	–	REF	–	–	REF	–	–
Overweight	1.02	(−0.58, 2.61)	0.21	1.094	(−0.56, 2.75)	0.195	0.05	(−0.01, 0.10)	0.098	0.05	(−0.01, 0.10)	0.106
Obesity	0.14	(−1.21, 1.49)	0.841	0.21	(−1.17, 1.59)	0.768	0.09	(0.05, 0.14)	<0.001	0.10	(0.05, 0.15)	<0.001
Model fit	*p*-value: <0.001; R-squared: 0.079			*p*-value: <0.001; R-squared: 0.110			*p*-value: <0.001; R-squared: 0.110			*p*-value: <0.001; R-squared: 0.117		

^a^Model 2 contains a subset of study participants excluding those who indicated race/ethnicity as “other” and modeling potential interaction of race/ethnicity and FRL status.

**Fig. 1:**
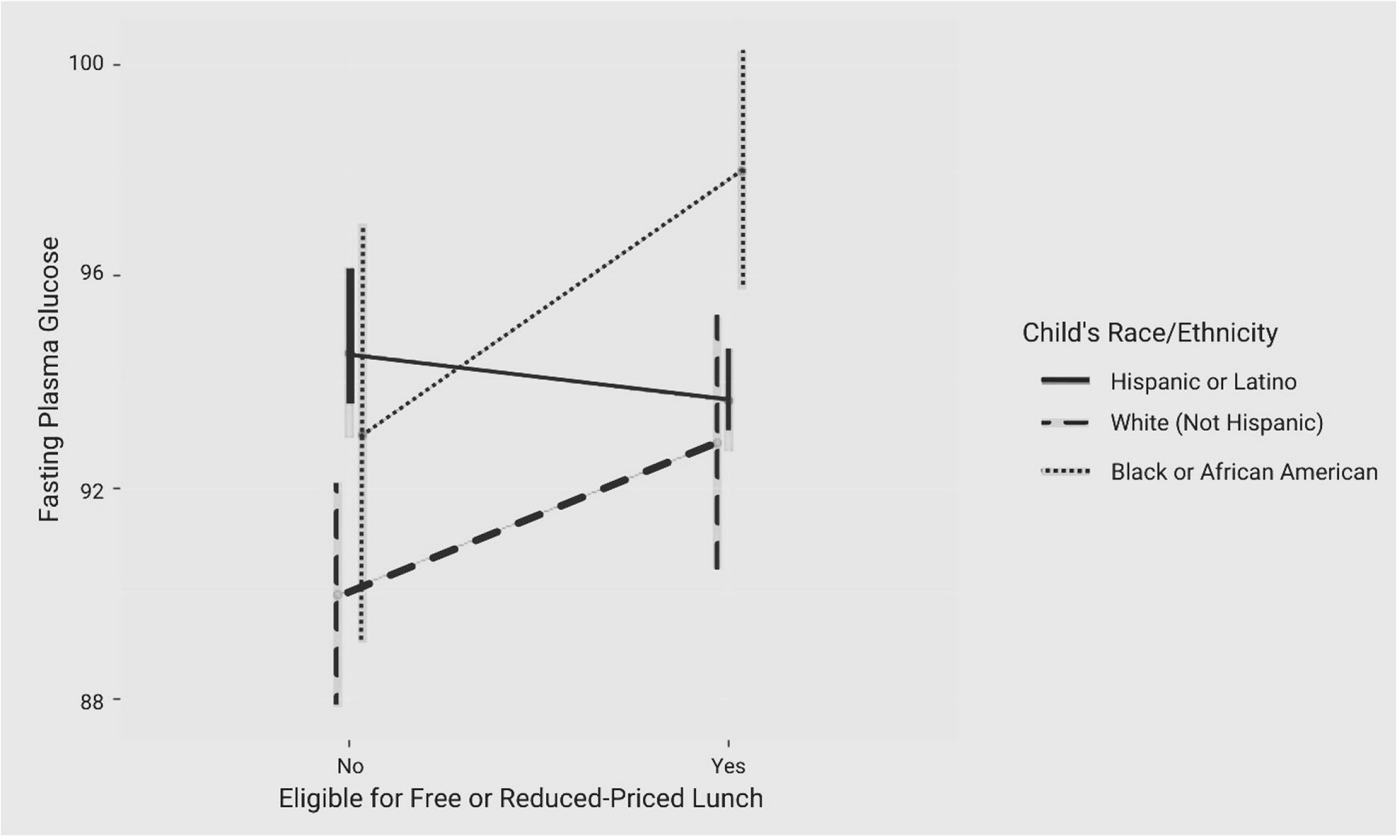
Interaction plot depicting interaction between child’s race or ethnicity, eligibility for free or reduced-price lunch, and fasting plasma glucose.

Model 2 assessed the relationship between the variables of interest and FPG and HbA1c and further modeled the interaction between race/ethnicity and FRL status on the outcome variables. There was a significant interaction between race/ethnicity and FRL status with respect to FPG (Table 2). Interestingly, Hispanic children eligible for FRL had a slightly lower FPG than those not eligible (mean 93.21 vs. 93.56). In contrast, among NHW children, those not eligible for FRL tended to have lower FPG than those eligible (mean 86.97 vs. 91.05). This result was similar among Black children (mean 90.04 vs. 95.93) (Fig. 1). There was no interaction effect of race/ethnicity and FRL status on HbA1c.

Table 3 reports the relationships between demographics and prediabetes status. The odds of having prediabetes were nearly two-fold higher in children ages 11–12 years compared to children ages 7–8 years (38% vs 27%, respectively, AOR = 1.85; 95%CI[1.01, 3.38], *p* = 0.047). The odds of having prediabetes were almost three- and five-fold higher in Hispanic and Black children compared to NHW children, respectively (AOR_Hisp_=2.82, 95%CI[1.53, 5.18], *p* = 0.001; AOR_Black_ = 4.93, 95%CI[2.42, 10.05], *p* < 0.001). Thirty percent of Hispanic children and 35% of Black children had prediabetes compared to 8% of NHW children. The odds of having prediabetes were over three-fold higher in children of parents with only an 8th-grade education (AOR = 3.12; 95%CI[1.69, 5.76], *p* < 0.001), nearly three-fold higher in children of parents who had some high school education (AOR = 2.83; 95%CI[1.52, 5.28], *p* = 0.001), and over two-fold higher in children of parents with a high school degree or GED (AOR = 2.25; 95%CI[1.31, 3.86], *p* = 0.003) compared to children of college graduates. Independent of demographics, the odds of having prediabetes were 1.5 times higher in children with obesity compared to those with underweight/normal weight (AOR = 1.50; 95%CI[1.07, 2.12], *p* = 0.02), with 31% of children with obesity having prediabetes, compared to 21.7% of children of normal weight. Sex and FRL status were not significant predictors for prediabetes.

**Table 3:** Binary logistic regression for the relationship between demographic variables and prediabetes status for TX Sprouts participants^a^.

	All TX Sprouts participants (*n* = 976)				Hispanic TX Sprouts participants (*n* = 461)			
	*n* (%) with prediabetes^b^	AOR	95% CI	*p*-value	*n* (%) with prediabetes^c^	AOR	95% CI	*p*-value
Sex								
Female	128 (27.6%)	REF	–	–	96 (28.7%)	REF	–	–
Male	128 (25.0%)	0.88	(0.84, 1.54)	0.392	101 (31.3%)	1.11	(0.78, 1.57)	0.562
Age (years)								
7 to 8	55 (26.7%)	REF	–	–	43 (31.6%)	REF	–	–
9	82 (23.4%)	0.88	(0.58, 1.32)	0.526	60 (26.3%)	0.79	(0.49, 1.27)	0.328
10	92 (26.4%)	0.98	(0.65, 1.47)	0.915	72 (29.4%)	0.94	(0.59, 1.49)	0.776
11 to 12	27 (38.0%)	1.85	(1.01, 3.38)	0.047	22 (44.9%)	1.81	(0.91, 3.63)	0.093
Race/ethnicity								
Non-Hispanic White	14 (8.4%)	REF	–	–	–	–	–	–
Hispanic	197 (29.9%)	2.82	(1.53, 5.18)	0.001	–	–	–	–
Black	34 (34.7%)	4.93	(2.42, 10.05)	<0.001	–	–	–	–
Other	11 (20.8%)	2.13	(0.88, 5.17)	0.094	–	–	–	–
Free/reduced-price lunch recipient								
No	51 (18.1%)	REF	–	–	42 (30.4%)	REF	–	–
Yes	205 (29.5%)	1.29	(0.88, 1.90)	0.192	155 (42.5%)	0.91	(0.59, 1.42)	0.688
Parent education								
College graduate	27 (13.4%)	REF	–	–	13 (17.6%)	REF	–	–
Some college/vocational school	43 (19.3%)	1.24	(0.71, 2.15)	0.452	26 (20.3%)	1.26	(0.59, 2.67)	0.555
High school grad/GED	64 (31.8%)	2.25	(1.31, 3.86)	0.003	136 (35.3%)	2.57	(1.26, 5.26)	0.010
Some high school	37 (37.4%)	2.83	(1.52, 5.28)	0.001	30 (37.0%)	2.85	(1.32, 6.18)	0.008
Finished 8th grade	42 (39.6%)	3.12	(1.69, 5.76)	<0.001	38 (38.0%)	3.02	(1.44, 6.34)	0.003
Less than 8th grade	42 (29.5%)	1.89	(1.03, 3.45)		42 (30.2%)	2.07	(1.00, 4.295)	0.050
BMI category								
Underweight/normal	106 (21.7%)	REF	–	–	77 (25.3%)	REF	–	–
Overweight	57 (30.5%)	1.4	(0.94, 2.07)	0.1	50 (36.0%)	1.55	(0.99, 2.42)	0.054
Obesity	93 (31.0%)	1.5	(1.07, 2.12)	0.02	70 (32.3%)	1.43	(0.96, 2.13)	0.078
	Model fit: *p*-value: <0.001 Nagelkerke R square: 0.123				Model fit: *p*-value: 0.002 Nagelkerke R square: 0.065			

^a^All variables were included in the binary logistic regression model together.

^b^256 of 976 children had prediabetes. This column displays percentages for those 256 within each variable’s categories.

^c^197 of 461 Hispanic or Latino children had prediabetes. This column displays percentages for those 197 within each variable’s categories.

When the sample was stratified by race/ethnicity, the subset of NHW children and Black children each had too few participants and events per value for reliable logistic regression analysis (NHW subset, *n* = 276 with 14 cases of prediabetes; Black subset, *n* = 94 with 34 cases of prediabetes) [22]. Among the Hispanic children (*n* = 461), 197 children had prediabetes (Table 3). The odds of having prediabetes were over three times as high in children of parents with only an 8th-grade education (AOR = 3.02; 95%CI[1.44, 6.34], *p* = 0.003), nearly three-fold higher in children of parents who had some high school education (AOR = 2.85; 95%CI[1.32, 6.18], *p* = 0.008), and over two-fold higher in children of parents with a high school degree or GED (AOR = 2.57; 95%CI[1.26, 5.26], *p* = 0.010) compared to Hispanic children whose parent graduated college (Table 3). Sex, age, FRL status, and BMI were not significant predictors for prediabetes in this subsample of only Hispanic children.

## Discussion

Hispanic and Black children had higher odds of having prediabetes than NHW children. Children of parents with no college education had significantly higher odds of having prediabetes than children of college graduates. Older children (11–12 years) had higher odds of having prediabetes compared to younger children (7–8 years), and children with obesity were significantly more likely to have prediabetes than those with underweight or normal weight. Among only Hispanic children, parent’s education status was the only significant predictor of prediabetes.

The current ADA guidelines for prediabetes testing in asymptomatic children recommend that youth, beginning after 10 years or at the onset of puberty (whichever occurs first), should be tested if they are overweight or obese and have at least one of the following four risk factors: (1) maternal history of diabetes or gestational diabetes mellitus during the child’s gestation, (2) family history of type 2 diabetes, (3) Native American, African American, Latino, Asian American, or Pacific Islander race/ethnicity, or (4) signs of insulin resistance or conditions associated with insulin resistance. There are no guidelines for testing for prediabetes in any prepubescent children before age 10 or in older children who do not have overweight or obesity [19]. However, this study’s findings highlight that prediabetes may be an undiagnosed issue in many children, especially those in more vulnerable minority communities and that more comprehensive guidelines may be needed.

In line with prior adolescent and adult literature, this study found that Hispanic and Black children were significantly more likely to have prediabetes than NHW children [11, 23]. Being of Hispanic or Black race/ethnicity is considered a risk factor for T2D [19]. Obesity is a well-established risk factor and precursor to T2D, and Hispanics and Black people of all ages also have higher rates of overweight and obesity compared to NHW people [1]. Hispanic adults have higher levels of visceral adiposity compared to their non-Hispanic peers, which can increase their risk of developing T2D [24]. Independent of adiposity, studies have also shown that Hispanic and Black adults have increased insulin resistance and higher acute insulin response compared to NHW adults [25, 26]. In Hispanic youth with overweight, increased insulin resistance and a higher acute insulin response have led to β-cell deterioration, which can lead to the development of T2D [27]. Black adolescents and adults may have impaired β-cell function compared to NHW [28, 29]. Meta-analyses found that Hispanic and Black adults with diabetes had higher HbA1c levels than their NHW counterparts and concluded that future research should focus on understanding the reasons behind these disparities [30, 31].

Socioeconomic status and social environments of minority groups may put them at increased risk of obesity and T2D compared to NHW groups. In the US, 17% of Hispanic people and 21% of Black people experienced poverty in the US, compared to just 9% of NHW people [32]. Poverty has been linked to increases in overweight and obesity among children [4], and the American Academy of Pediatrics states that poverty during childhood can lead to an increase in adverse health outcomes spanning through adulthood [5]. Individuals living in poverty in the US may be disproportionately affected by obesity due to the obesogenicity of their environments, defined as the sum of physical, economic, political, and sociocultural that promote obesity [33]. Obesogenic environments are often marked by a lack of access to fresh foods, safe neighborhood parks or sports facilities [34], and healthcare services, and such environments have been associated with increased odds of having diabetes [35].

In this study, there were no differences in prediabetes rates between children who received FRL at school and those who did not. However, approximately 70% of children in this study were receiving FRL, so the homogeneity of the population may explain these null findings. In addition, most of the children who participated in the study lived in the neighborhoods zoned for the participating schools; therefore, the obesogenicity of the environment was likely similar for all enrolled children.

Another component of socioeconomic status that contributes to health is educational attainment, which is often inversely correlated with poverty rates [4]. On average, Hispanic and Black individuals tend to have lower educational attainment than their NHW counterparts [7]. While poverty was not significantly associated with prediabetes in this study, the odds of child prediabetes decreased significantly as parent educational attainment increased. This was particularly pronounced among Hispanic children, who had more than twice the risk of prediabetes if their parents did not attend college compared to other Hispanic children whose parents graduated college.

This is consistent with previous research that found a positive relationship between greater educational attainment and health status. Low educational attainment is considered a predictor of low health literacy [33] and may therefore affect individuals’ ability to find, understand, and use information and services to make health decisions [36]. A review study by Lazar et al. concluded that the most substantial barrier to access to healthcare for children from lower-income households is a lack of parent education [37]. Potential issues for children of parents with low educational attainment include that their regular well-child visits may not be prioritized, risk of health issues may not be perceived, physical activity and healthy food choices may not be encouraged, and medical instructions or information may be misunderstood [37]. These findings suggest that parent educational attainment has the potential to serve as a useful screening demographic to increase health literacy and decrease subsequent obesity and prediabetes rates in children at risk.

In this study, older children (11–12 years) had a higher prevalence of prediabetes compared to younger children (7–8 years). This may be due to the physical changes that occur with age; as children reach puberty, insulin resistance increases [38], which can, in turn, impact glucose tolerance and increase FPG and HbA1c levels. Although there was a positive association between overweight and obesity rates and age in this study, prediabetes rates were higher in older compared to younger children even after adjusting for BMI, which suggests that obesity status is not the sole driving factor in the association between age and prediabetes. The well-known effects of puberty on insulin resistance do not temper the concerns raised by the prevalence of prediabetes among older children in this study because Black and Hispanic children revert to normal glucose levels after puberty at lower rates than NHW children [13].

Being overweight or obese is considered a main risk factor for T2D and is a testing requirement based on current ADA guidelines, which only recommend testing a child if they are overweight or obese and have at least one other risk factor [19]. This study found that independent of age and other demographic characteristics, children with obesity were more likely to have prediabetes than those with underweight or normal weight, suggesting that obesity alone can be a predictor of T2D, even in the absence of other risk factors such as race/ethnicity. This observation is consistent with prior research finding a linear relationship between an increasing BMI through puberty and progression to T2D [10]. However, among Hispanic children, neither overweight nor obese significantly increased the odds of having prediabetes, and over one-fourth of Hispanic children who had a normal or underweight BMI had prediabetes. This suggests that the threshold BMI requirements for prediabetes screening may need to be relaxed in this population.

There are currently no guidelines for testing prediabetes in prepubescent children under age 10 or in pubescent children without both overweight or obesity and at least one other risk factor. In the development of more inclusive testing recommendations for children, ADA should consider creating diagnostic cutoffs specifically for children rather than using the adult criteria to diagnose children. Some scientists have suggested that the diagnostic tests and cutoffs based on adults may not be equally effective or accurate in pediatric populations and that the development of T2D in children may be more accelerated than in adults [39–41]. Child-designed cutoffs, in combination with more inclusive guidelines and screening in children, would allow future pediatric research to establish accurate prediabetes prevalence rates and develop prevention programs accordingly.

Over a quarter of the children in this study had prediabetes. It is important to note that the parents of these children did not actively seek diabetes testing for them but instead capitalized on the opportunity provided by this study for a convenient and free diabetes test with a cash incentive at their children’s schools. Although the majority of the children in this study did have at least one risk factor for diabetes (based on race, ethnicity, and/or BMI), they were likely not being screened otherwise. The high prediabetes rates in this study emphasize the need for young children to be tested more regularly. One way to screen children more frequently and at a higher rate would be to include FPG and/or HbA1c testing at their annual check-ups. This would allow for asymptomatic children who do not meet the testing requirements to have their diabetes risk checked. Another method could be to have community programs that provide free diabetes screenings for children at schools or community centers once or twice per year.

This study has many strengths. This is the first study to collect FPG and HbA1c on such a large sample of nearly 1000 children below 12 years of age. In addition, it examines a high-risk and vulnerable group—children from minority backgrounds experiencing socioeconomic disadvantage—giving insight into the health of these groups. It is also conducted in a non-healthcare school setting. While smaller studies tend to examine solely children who are obese or who already have a diabetes diagnosis or signs and symptoms of diabetes in a healthcare setting, this study examined all children in their more representative natural settings without specifically targeting those at risk. This creates a stronger understanding of the current prevalence in this population, which is currently lacking in the literature.

This study also has a few limitations. First, given that the sample is primarily children experiencing socioeconomic disadvantage, it is difficult to generalize results to a population of different socioeconomic statuses. Future research should explore the findings of this analysis in a larger, nationally representative sample. Fasting also presents a slight limitation; although parents were reminded through text messages and flyers and children are asked three times about fasting on the morning of the blood draw, the chance of a child eating before their blood draw remains a possibility. In addition, blood was only collected at one time point, which may not have been representative of that child’s usual blood sugar levels. However, HbA1c was added in wave two of the study to provide a more long-term measure of glycemic control. This study also did not collect data on pubertal status, which could give insight into the theories behind the associations of age and prediabetes. Additionally, the study did not collect data on social determinants of health that could explain the causes of some of the racial disparities highlighted in this study. Relatedly, this study used cross-sectional analyses; therefore, no causal relationships can be established.

Prediabetes may be a larger and more widespread issue in young children than the literature has previously established. Given that Hispanic and Black populations in the US are at increased risk for obesity and diabetes and struggle with higher poverty rates and lower educational attainment than NHW populations, early screening is essential in the prevention of T2D in these high-risk populations. Early screening can lead to early prevention and treatment options for this high-risk population.

## Data Availability

The datasets generated during and/or analyzed during the current study are not publicly available to preserve the confidentiality of minor participants but are available from the corresponding author upon reasonable request.
